# Integrating machine learning and single‐cell analysis to uncover lung adenocarcinoma progression and prognostic biomarkers

**DOI:** 10.1111/jcmm.18516

**Published:** 2024-07-03

**Authors:** Pengpeng Zhang, Jiaqi Feng, Min Rui, Jiping Xie, Lianmin Zhang, Zhenfa Zhang

**Affiliations:** ^1^ Department of Lung Cancer, Tianjin Lung Cancer Center, National Clinical Research Center for Cancer, Key Laboratory of Cancer Prevention and Therapy, Tianjin's Clinical Research Center for Cancer Tianjin Medical University Cancer Institute and Hospital Tianjin China; ^2^ Department of Pathology Tianjin Medical University Cancer Institute and Hospital, National Clinical Research Center for Cancer, Tianjin's Clinical Research Center for Cancer Tianjin China

**Keywords:** immunotherapy response, lung adenocarcinoma, machine learning, S100A16, single‐cell analysis

## Abstract

The progression of lung adenocarcinoma (LUAD) from atypical adenomatous hyperplasia (AAH) to invasive adenocarcinoma (IAC) involves a complex evolution of tumour cell clusters, the mechanisms of which remain largely unknown. By integrating single‐cell datasets and using inferCNV, we identified and analysed tumour cell clusters to explore their heterogeneity and changes in abundance throughout LUAD progression. We applied gene set variation analysis (GSVA), pseudotime analysis, scMetabolism, and Cytotrace scores to study biological functions, metabolic profiles and stemness traits. A predictive model for prognosis, based on key cluster marker genes, was developed using CoxBoost and plsRcox (CPM), and validated across multiple cohorts for its prognostic prediction capabilities, tumour microenvironment characterization, mutation landscape and immunotherapy response. We identified nine distinct tumour cell clusters, with Cluster 6 indicating an early developmental stage, high stemness and proliferative potential. The abundance of Clusters 0 and 6 increased from AAH to IAC, correlating with prognosis. The CPM model effectively distinguished prognosis in immunotherapy cohorts and predicted genomic alterations, chemotherapy drug sensitivity, and immunotherapy responsiveness. Key gene S100A16 in the CPM model was validated as an oncogene, enhancing LUAD cell proliferation, invasion and migration. The CPM model emerges as a novel biomarker for predicting prognosis and immunotherapy response in LUAD patients, with S100A16 identified as a potential therapeutic target.

## 1 INTRODUCTION

Lung cancer (LC) is a common malignant tumour and one of the leading causes of cancer‐related deaths worldwide.^1^ It is typically classified into non‐small cell lung cancer (NSCLC) and small cell lung cancer (SCLC), with NSCLC accounting for approximately 85% of all LC cases. Among NSCLCs, lung adenocarcinoma (LUAD) is the most prevalent subtype.^2^ Many patients with LUAD have a poor prognosis because they are diagnosed at an advanced stage, underscoring the importance of improving early detection rates to extend patient survival.^3^

The development of LUAD can be broadly divided into four stages: atypical adenomatous hyperplasia (AAH), adenocarcinoma in situ (AIS), minimally invasive adenocarcinoma (MIA), and invasive adenocarcinoma (IAC).^4^ Studies have shown that surgical resection of tumours in the first three stages results in nearly 100% 10‐year recurrence‐free survival and overall survival (OS) rates. However, the prognosis significantly declines when LUAD progresses to the IAC stage, which is the most common form found in postoperative pathology.^5^, ^6^ Traditional methods are not effective in accurately identifying the stage of LUAD. While thin‐section CT scanning and low‐dose CT can detect small early‐stage LUADs, evaluating the stage of LUAD based solely on radiographic parameters remains challenging.^7^ Moreover, preoperative biopsies pose risks of localization difficulties and sampling failures.^8^, ^9^ Single‐cell sequencing technology can reveal the molecular mechanisms of cancer development at the genetic level, identify diagnostic and prognostic markers, and has been widely used in tumour research. This technology may provide early support for staging LUAD.^10^, ^11^

The biological mechanisms underlying the evolution of LUAD are still unclear, and the key factors driving tumour progression and markers for identifying tumour staging are poorly understood.^12^ Traditional research has mostly focused on the bulk level, using genomic, transcriptomic and proteomic approaches to understand the development of premalignant lesions into cancer.^13^ However, detailed cell populations and genes involved in the invasive progression of LUAD from AAH to IAC remain largely unknown.^14^, ^15^ Single‐cell sequencing technology offers a higher resolution analytical tool that allows researchers to observe cancer at the molecular level, offering a deeper understanding of LUAD.^16^

This study integrated three high‐throughput single‐cell sequencing datasets from patients with LUAD. Malignant epithelial cells were extracted and classified into AAH, AIS, MIA and IAC based on their progression, exploring the heterogeneity of different stages of cancer tissues in LUAD. The study aimed to identify risk factors influencing the continuous progression of LUAD and to discover biomarkers aiding in identifying LUAD staging.

## 2 METHOD

### 2.1 Dataset source

For the analysis, two Single‐Cell RNA Sequencing (scRNA‐seq) datasets were sourced from the Gene Expression Omnibus (GEO) database (GSE150938 and GSE189357, http://www.ncbi.nlm.nih.gov/geo), and another from the Genome Sequence Archive (GSA) in the BIG Data Center (HRA001130). LUAD transcriptomic, methylation, copy number variation (CNV), mutation, and clinical data were successfully retrieved from The Cancer Genome Atlas (TCGA) database (https://portal.gdc.cancer.gov). Six transcriptome datasets for model validation were also acquired from GEO, including GSE13213^17^ (*n* = 119), GSE26939^18^ (*n* = 115), GSE29016^19^ (*n* = 39), GSE30219^20^ (*n* = 86), GSE31210^21^ (*n* = 227) and GSE42127^22^ (*n* = 134). Additionally, 296 cases of immunotherapy‐treated LUAD were analysed from OAK and POPLAR, two major clinical trials focusing on chemotherapy and immunotherapy for NSCLC. To ensure the uniformity and comparability of the data, gene expression data were first converted into transcripts per million (TPM) format. Following this, the ‘combat’ function from the ‘sva’ package was utilized to address potential batch effects. Additionally, log transformation was carried out on all datasets sourced from both TCGA and GEO databases, thus establishing a standardized data format from the outset of the analysis.

### 2.2 Single‐cell RNA sequencing data analysis

The initial single‐cell gene expression matrix underwent preprocessing utilizing the Seurat R package (version 4.2.0). Inclusion criteria for genes mandated expression in a minimum of 10 cells. Quality control measures led to the exclusion of cells with either more than 5000 or fewer than 200 expressed genes, or those with over 10% of their unique molecular identifiers (UMIs) originating from mitochondrial gene. These steps resulted in a refined single‐cell transcriptomic expression matrix. Batch effects were addressed through integration using the Harmony R package. Dimensionality reduction to visualize the data were achieved through t‐distributed Stochastic Neighbour Embedding (t‐SNE). The ‘FindAllMarkers’ function facilitated the identification of differentially expressed genes (DEGs) across each cellular subpopulation.

### 2.3 Analysing tumour cell developmental trajectories and metabolic pathway activity

The Monocle2 algorithm was deployed for developmental trajectory analysis on inferred tumour cells, utilizing a gene‐cell matrix derived from UMI counts, which was normalized within a subset of Seurat. A novel ‘cell data set’ function was employed to create an object, setting the expression family parameter to the Negative Binomial distribution size. Following dimensionality reduction and ordering of units, cell trajectories were deduced using standard parameters. The CytoTRACE package^23^ was utilized to evaluate the stemness and differentiation potential across various tumour cell subpopulations. Furthermore, the scMetabolism package^24^ was employed to assess metabolic pathway activity within distinct subtypes of tumour epithelial cells.

### 2.4 Identifying key prognostic signatures in LUAD using machine learning algorithms

The GSVA^25^ package was utilized to determine the prevalence of specific tumour clusters in LUAD specimens. A univariate Cox regression analysis assessed the influence of pivotal genes within these clusters on LUAD patient survival. Following this, a comprehensive evaluation employing 10‐fold cross‐validation was conducted, incorporating a suite of 10 machine learning algorithms, including stepwise Cox, Lasso, Ridge, Cox partial least squares regression (plsRcox), CoxBoost, random survival forest (RSF), Generalized Boosted Regression Models (GBM), Elastic Net (Enet), Supervised Principal Components (SuperPC) and Survival Support Vector Machine (survival‐SVM). This methodology aimed to pinpoint the most critical prognostic signature, distinguished by the highest concordance index (C‐index).

### 2.5 Analysing immune cell composition

Seven diverse algorithms for assessing immune cell infiltration—EPIC, TIMMER, CIBERSORT, CIBERSORT‐ABS, MCPCounter, QUANTISEQ and XCELL—were applied to evaluate the immune cell composition. Furthermore, the estimate package^26^ was strategically used to calculate immune, stromal and ESTIMATE scores for patients with TCGA‐LUAD, facilitating an in‐depth analysis of the tumour microenvironment (TME).

### 2.6 Cultivation of human lung adenocarcinoma cell lines

A549 and H1299, human LUAD cell lines, were acquired from the Shanghai Life Sciences Institute's Cell Resource Center. These cells were propagated in either F12K or RPMI‐1640 medium (Gibco BRL, USA), enriched with 10% fetal bovine serum (FBS) and 1% antibiotics (streptomycin and penicillin from Gibco, Invitrogen, Waltham, MA, USA). Cultivation was performed at 37°C in an atmosphere containing 5% CO_2_ and 95% relative humidity.

### 2.7 Silencing of S100A16 via siRNA transfection

To knock down S100A16 expression, small interfering RNA (siRNA) targeting S100A16 (siS100A16) was utilized alongside a negative control (NC) siRNA. Cells were plated at a density ensuring 50% confluency in 6‐well plates and transfected using Lipofectamine 3000 (Invitrogen, USA) following the manufacturer's instructions.

### 2.8 Colony formation ability post‐transfection

For colony formation assays, 1 × 10^3^ transfected cells were plated per well in 6‐well plates and cultured for 14 days. Following culture, cells were fixed with 4% paraformaldehyde for 15 min and stained with crystal violet (Solarbio, China) after being washed twice with PBS.

### 2.9 Monitoring cell migration via wound‐healing assay

To assay cell migration, transfected cells were grown in 6‐well plates until 95% confluency was reached. A sterile 20 μL pipette tip was used to create a uniform scratch across the cell monolayer. After scratching, cells were rinsed twice with PBS to remove detached cells. Wound closure was documented at 0 and 48 h post‐scratch using ImageJ software for scratch width measurement.

### 2.10 Evaluating cell invasion and migration with transwell assay

The transwell assay was employed to examine the invasive and migratory properties of A549 and H1299 cells post‐treatment. Cells (2 × 10^5^) were placed in the top chamber of 24‐well plates, with or without a Matrigel coating, for 48 h. Post‐incubation, non‐invading cells were removed from the top layer, while cells that migrated to the bottom were fixed with 4% paraformaldehyde and stained with 0.1% crystal violet (Solarbio, China).

### 2.11 Statistical methodologies for data analysis

Statistical analyses, data manipulation and graphical representations were carried out using R software, version 4.2.0. The Kaplan–Meier estimator and log‐rank test were employed to compare OS among different subtypes. For assessing variance in continuous data between groups, either the Wilcoxon rank‐sum test or Student's *t*‐test was used. Categorical data analysis utilized either the chi‐squared test or Fisher's exact test. To account for multiple testing, *p*‐values were adjusted using the false discovery rate (FDR) method. Pearson's correlation coefficient was employed to explore variable relationships. All statistical analyses were two‐sided, with a significance level set at *p* < 0.05.

## 3 RESULTS

### 3.1 Cell classification and CNV analysis

**FIGURE 1:**
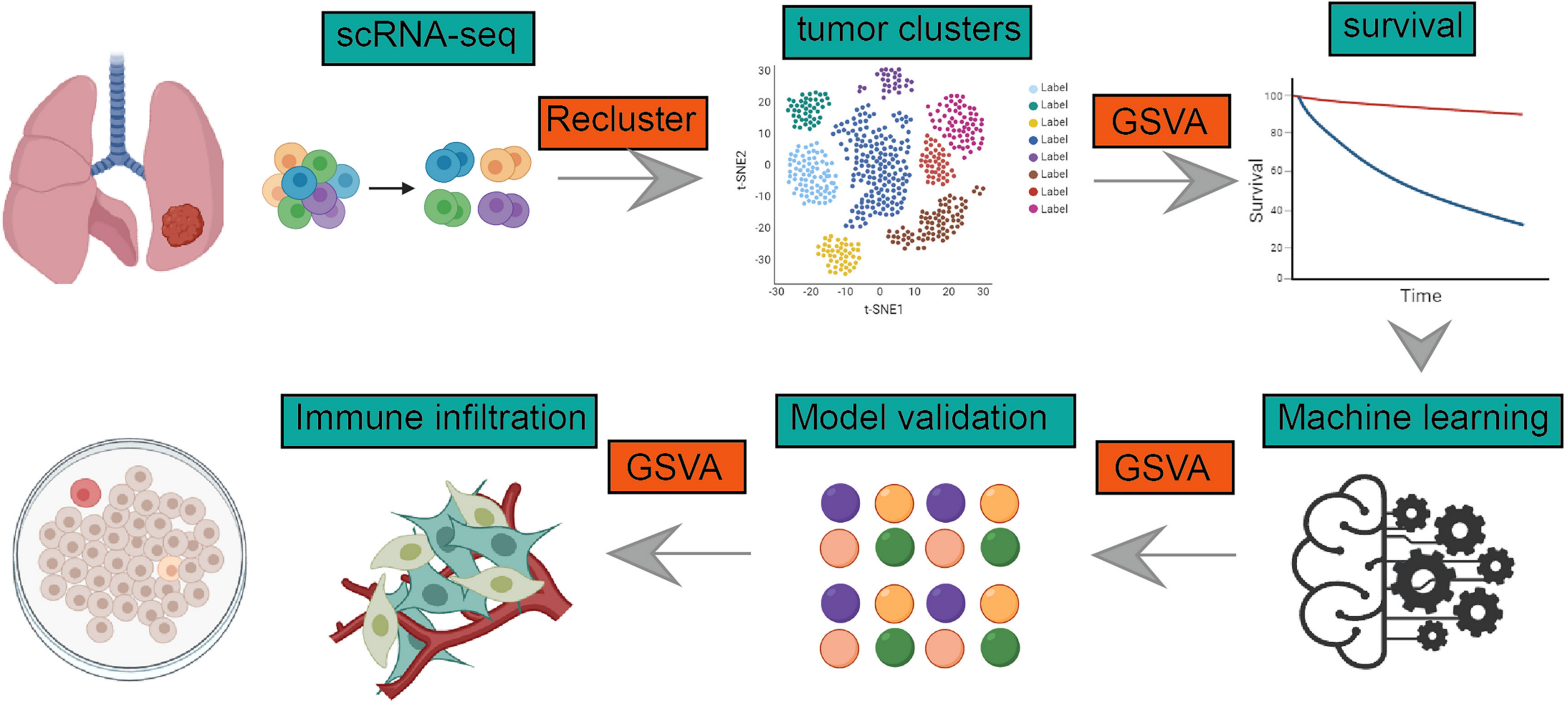
Utilizing single‐cell data, we analysed the heterogeneity of different subgroups of tumour cells in lung adenocarcinoma and constructed a model using machine learning. Ultimately, through experimental validation, S100A16 was identified as a potential therapeutic target for lung adenocarcinoma.

**FIGURE 2:**
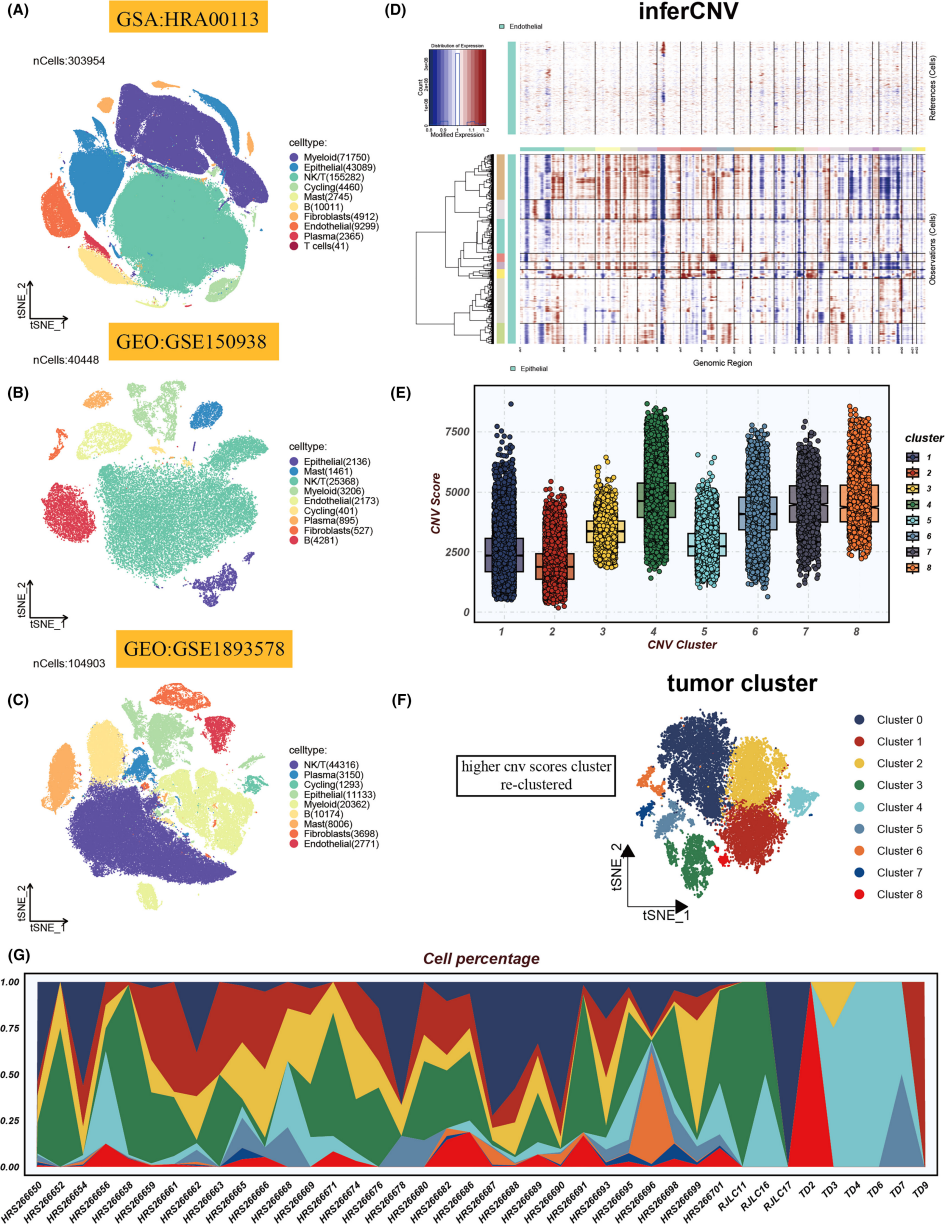
Cellular heterogeneity and genomic alterations in single‐cell analyses. (A–C) t‐distributed stochastic neighbour embedding (tSNE) visualizations highlight cell type distributions within HRA00113, GSE150938 and GSE189357 scRNA‐seq cohorts. (D) A heatmap delineates cell‐wise genomic copy number variations (CNVs), calculated from gene expression proximal to chromosomal loci, with amplifications in red and deletions in blue. (E) Box plots reveal CNV patterns across eight identified clusters. (F) tSNE plot illustrates the spatial distribution of tumour subgroups. (G) The relative abundance of nine tumour cell clusters across various samples is depicted.

Figure 1 outlined the workflow for the entire analysis. Canonical marker genes (Figure S1A–C) were utilized to classify all sampled cells from datasets GSE150938, GSE189357 and HRA001130 into various cell types, as depicted in the t‐SNE plots (Figure 2A–C). Epithelial and endothelial cells from all datasets were extracted for reference, and a comprehensive analysis of CNVs across every chromosome in all cells was conducted using the InferCNV algorithm, as shown in Figure 2D. The analysis revealed that the majority of epithelial cells exhibited higher levels of CNVs compared to endothelial cells. Figure 2E displayed the variance in CNV scores across eight identified cell clusters, with Clusters 1 and 2 showing relatively lower CNVs, thus being designated as normal cells, while the remaining clusters were categorized as tumour cells. Subsequently, all tumour cells underwent re‐clustering. This process led to the identification of nine distinct clusters, as illustrated in Figure 2F, highlighting the varying abundance of different tumour cell clusters across samples (Figure 2G).

### 3.2 Dynamic gene expression and pathway enrichment in tumour cell clusters

**FIGURE 3:**
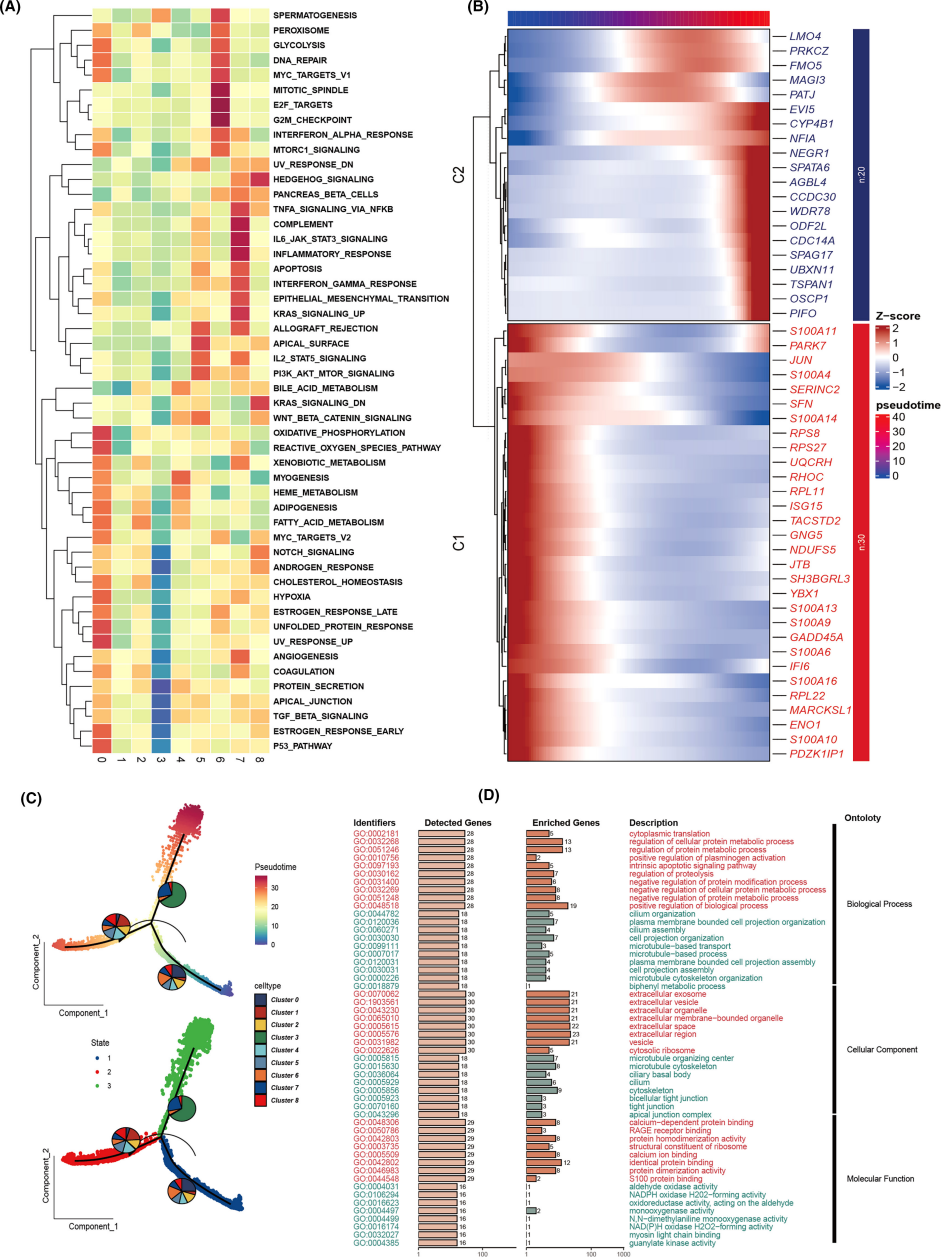
Decoding tumour cell cluster dynamics. (A) Enrichment analysis across distinct tumour cell clusters using Gene Set Variation Analysis (GSVA), visualized through a heatmap. (B) Expression dynamics across pseudotime are depicted in a heatmap, showcasing gene expression intensity variations. (C) Developmental trajectories of various tumour cell clusters are illustrated via pseudotime analysis, with cells colour‐coded based on tumour clusters or progression through pseudotime. (D) Gene ontology (GO) enrichment analysis identifies and highlights enriched pathways in genes from Clusters 1 and 2 as shown in (B), covering aspects of biological process (BP), cellular component (CC) and molecular function (MF).

The enrichment analysis of nine tumour cell clusters in key gene sets, as shown in Figure 3A, reveals a notable pattern. Specifically, the sixth group of cells demonstrates significant enrichment in biological pathways related to cell cycle processes and DNA repair mechanisms. Additionally, this cell cluster exhibits strong activity in the MYC target pathways V1 and V2, highlighting the potential pivotal role of MYC in driving the transcriptional programs of these tumour cells (Figure 3A). The heatmap delineates the gene expression profiles along a pseudotemporal continuum, as depicted in Figure 3B. Pseudotime mapping delineates the ontogenetic evolution of disparate neoplastic clusters. Intriguingly, Clusters 0 and 6 localize to the nascent stages of development, with a subsequent diminution in prevalence over time, as illustrated in Figure 3C. This trend may be indicative of an inherent propensity for stemness and differentiation potential within Clusters 0 and 6. Following this, GO enrichment analysis of pseudotime‐correlated genes underscores the salient pathways enriched across biological processes, cellular constituents and molecular functionalities, as delineated in Figure 3D. Red font underscores the enrichment of genes that are overexpressed in the initial stages, predominantly implicated in the cell cycle, DNA repair and protein metabolic processes. In contrast, green font details the enrichment of genes that are overexpressed in the culminating stages, primarily involved in biological processes such as ‘organelle organization’, ‘exosome’ and ‘extracellular vesicle’ pathways.

### 3.3 Metabolic profiling and stemness potential of tumour cell clusters

**FIGURE 4:**
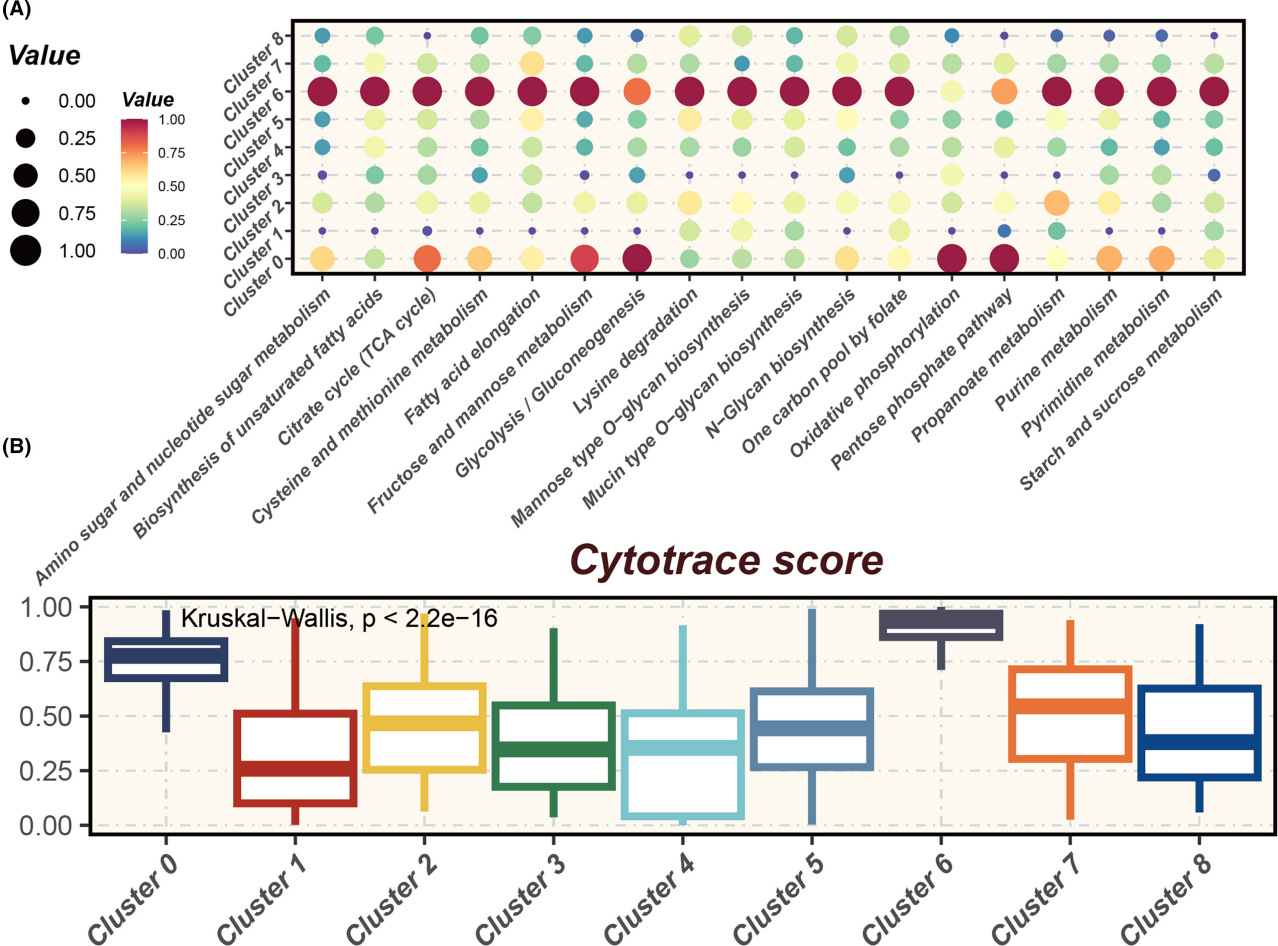
Metabolic heterogeneity and stemness potential across tumour clusters. (A) Bubble chart illustrating metabolic heterogeneity across various tumour clusters, highlighting differential metabolic activity within the tumour microenvironment. (B) Cytotrace analysis depicting Cytotrace scores for different tumour clusters, where higher scores indicate cells with greater stemness and differentiation potential.

Figure 4A illustrates the metabolic activity levels in distinct tumour cell clusters, ranging from Cluster 0 to Cluster 8. Notably, Cluster 6 shows significantly heightened activity in metabolic pathways such as amino sugar and nucleotide metabolism, glycolysis, gluconeogenesis, the citric acid cycle, glycerophosphate metabolism, and several amino acid metabolism pathways. This metabolic profile may be associated with Cluster 6's biological characteristics, including proliferative capacity, response to environmental stress, and potential stem cell‐like properties. Importantly, there is a significant statistical variance in Cytotrace scores between groups, with Cluster 0 and 6 exhibiting the highest stemness (Figure 4B), supporting their potential for enhanced proliferation and differentiation capabilities, consistent with previous pseudotime analyses.

### 3.4 Correlation between tumour cell cluster abundance and survival

**FIGURE 5:**
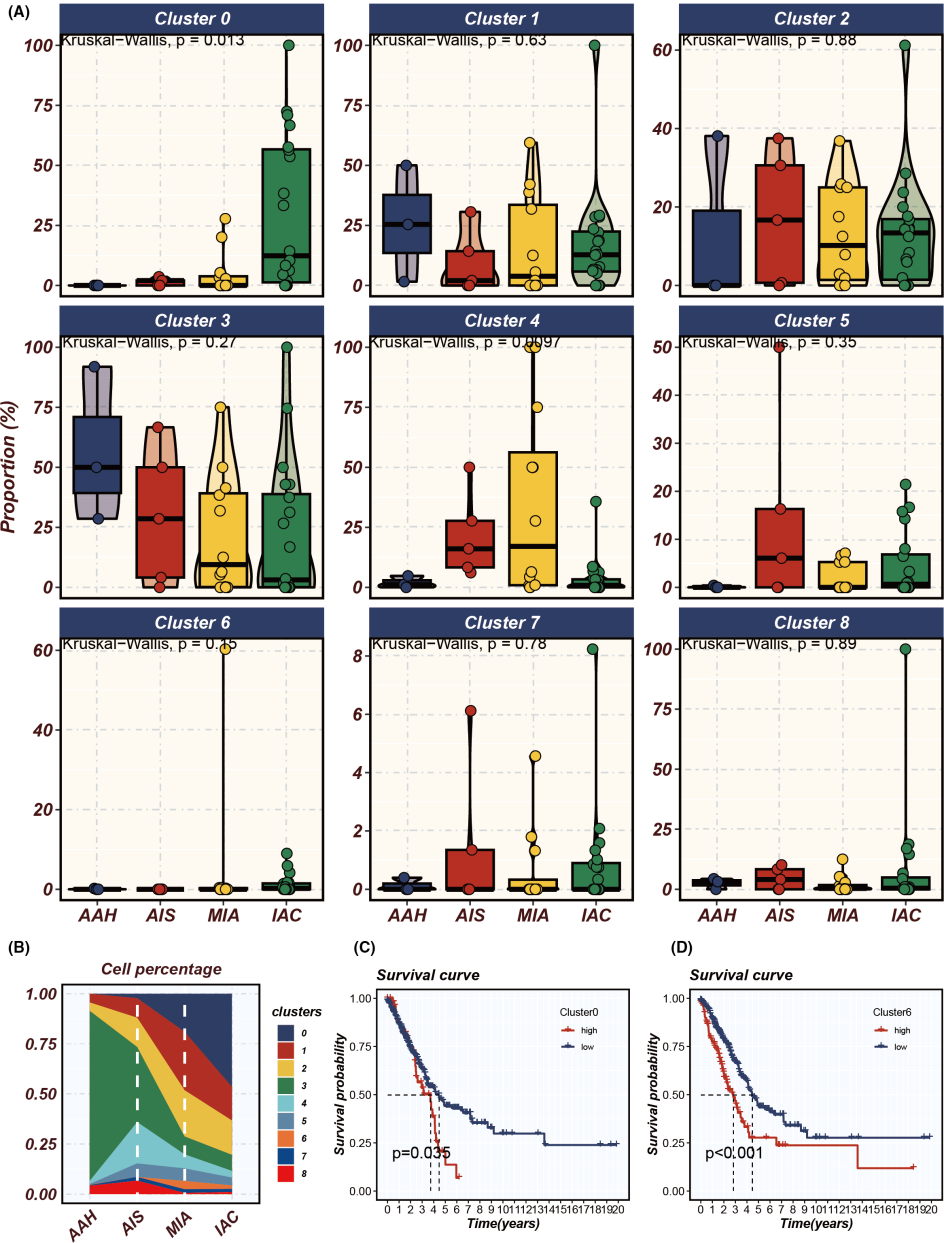
Analysing tumour cluster dynamics and survival impact in LUAD progression. (A) Proportional variations of different tumour clusters throughout the progression of LUAD (from atypical adenomatous hyperplasia [AAH] to adenocarcinoma in situ [AIS], minimally invasive adenocarcinoma [MIA] and finally to invasive adenocarcinoma [IAC]). (B) The prevalence of distinct tumour cell clusters during the progression stages. (C, D) Single‐sample Gene Set Enrichment Analysis (ssGSEA) assessing the impact of the abundance of Clusters 0 and 6 on the survival of patients with LUAD, where higher abundance indicates poorer prognosis.

Figure 5A delineates the cellular proportions within each cluster corresponding to distinct histopathological patterns: AAH, AIS, MIA and IAC. Notably, Clusters 0 and 4 exhibit variability in cell abundance across these histopathological states, with Cluster 0 showing an incremental increase in abundance with advancing malignancy. Complementarily, Figure 5B corroborates the progressive rise of Cluster 0 through the stages of malignancy, with a similar trend observed in Cluster 6. Consequently, leveraging the single‐sample Gene Set Enrichment Analysis (ssGSEA) algorithm, we quantitatively assessed the abundance of Clusters 0 and 6 in TCGA LUAD samples. Intriguingly, patients with a higher abundance of these clusters demonstrated poorer survival outcomes (Figure 5C,D). This suggests a potential prognostic relevance of these clusters in the context of LUAD progression and patient survival.

### 3.5 Development of a prognostic and immunotherapy‐related signature using machine learning algorithms

**FIGURE 6:**
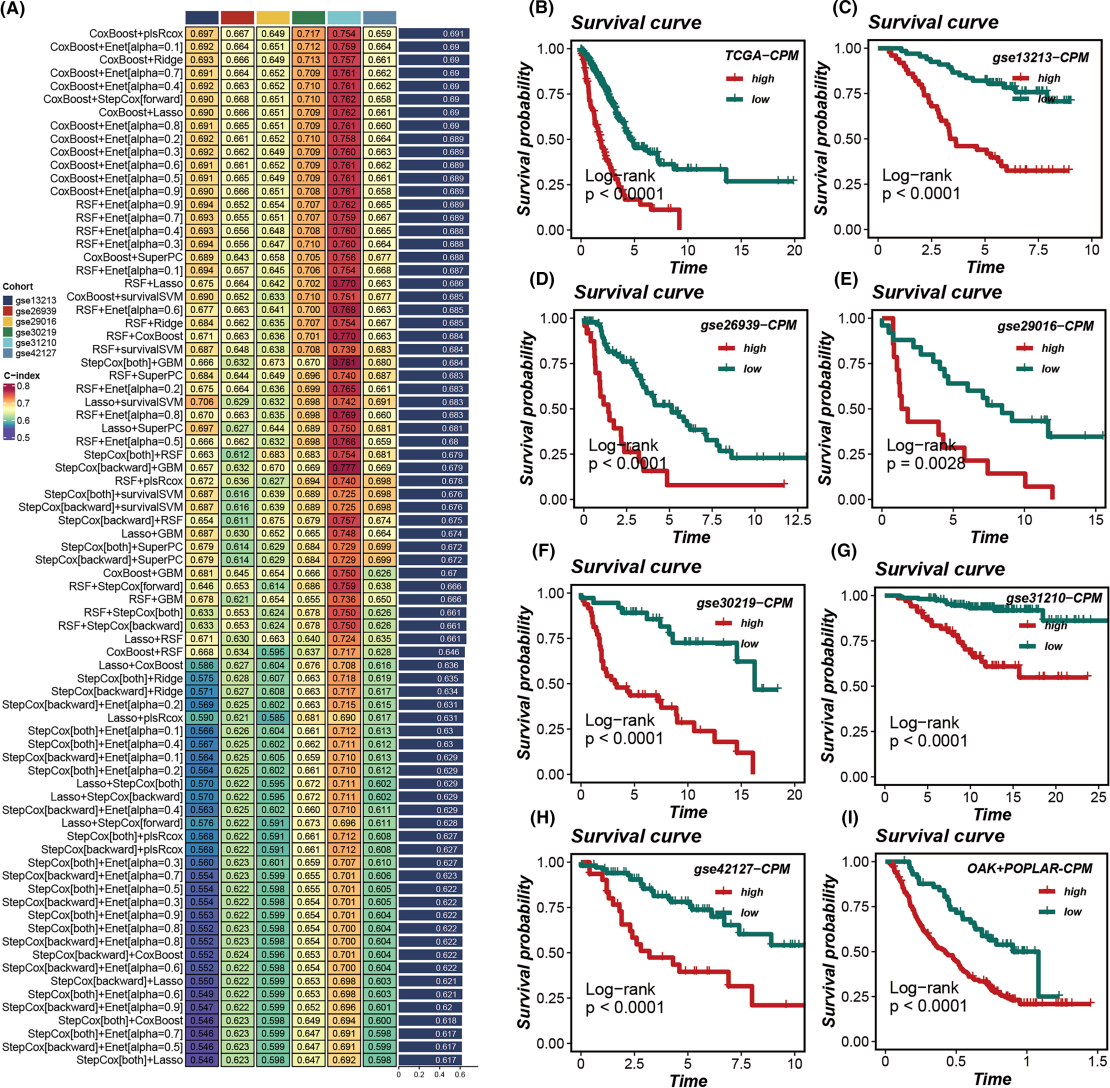
Construction and validation of the prognostic model. (A) Development of the prognostic model utilizing 10 machine learning approaches, with the concordance index (C‐index) serving as the evaluation metric; the CoxBoost and plsRcox algorithms were identified as the superior composite prognostic model (CPM). (B–H) Survival curves for patients categorized into high versus low CPM groups across seven cohorts, with *p*‐values determined using the log‐rank method to assess statistical significance. (I) Calculation of CPM scores within the immunotherapy cohort using the model's formula, followed by an assessment of their prognostic relevance.

Leveraging the marker genes from tumour cell Clusters 0 and 6, we have devised a prognostic and immunotherapy‐related signature utilizing a machine learning composite algorithm. The TCGA dataset was employed as the training cohort, with six GEO datasets utilized for validation purposes. The criterion for model selection was based on the average c‐index across the six validation cohorts. Ultimately, the CoxBoost and plsRcox algorithms were selected as the optimal composite prognostic model (CPM) (Figure 6A). The CPM score successfully stratified patient prognoses across all seven cohorts (Figure 6B–H), with patients in the high CPM group demonstrating poorer survival outcomes compared to those in the low CPM group. Additionally, extrapolating the CPM scores to the immunotherapy cohort using the model's formula revealed that the CPM scores continued to effectively discriminate prognostic outcomes (Figure 6I), suggesting its potential utility in predicting responses to immunotherapy treatments.

### 3.6 Superior prognostic efficacy of the CPM across multiple datasets

**FIGURE 7:**
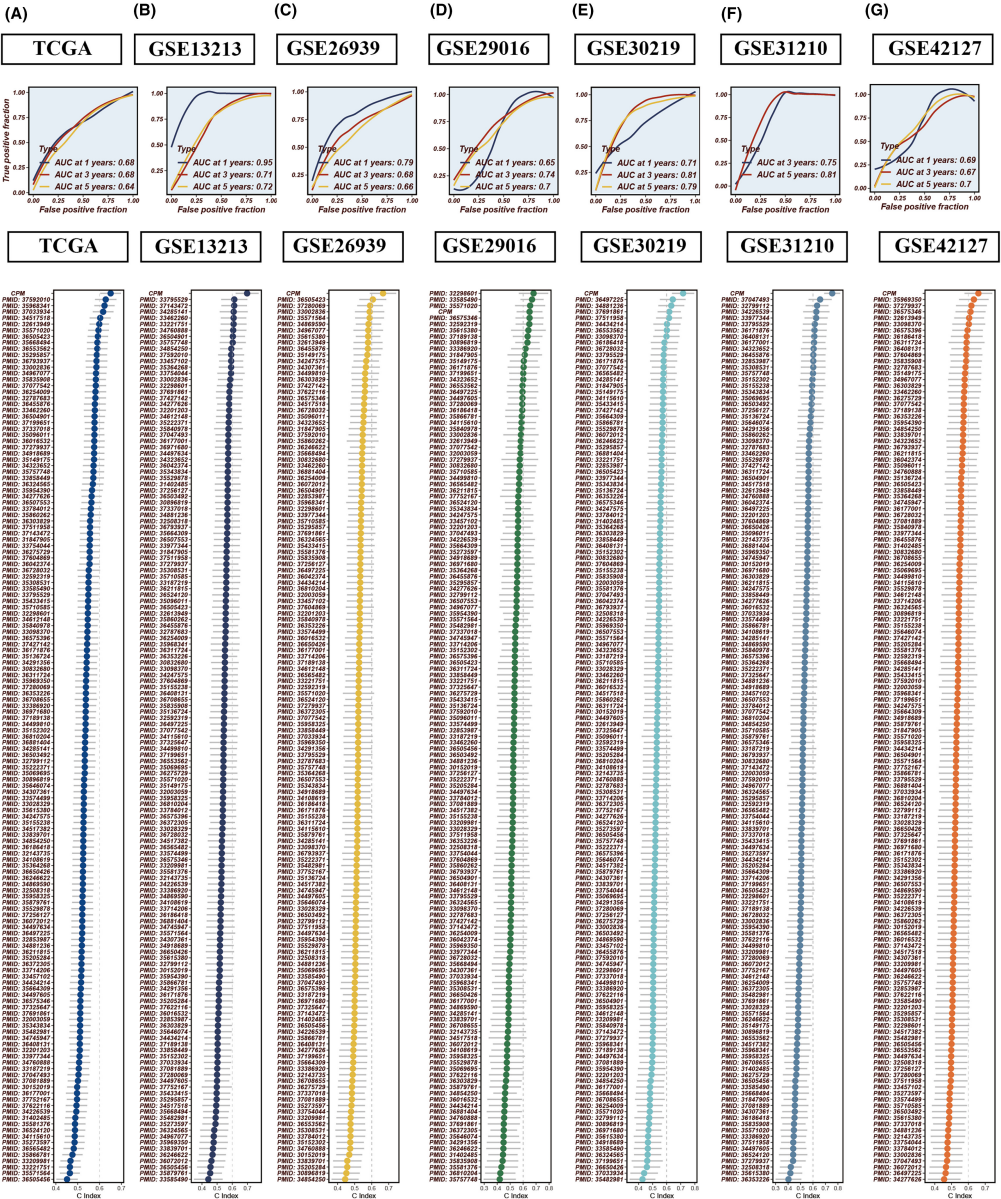
Comparative prognostic performance of CPM against established models. (A–G) receiver operating characteristic (ROC) curves evaluating the CPM within the TCGA, GSE13213, GSE26939, GSE29016, GSE30219, GSE31210 and GSE42127 LUAD datasets. When benchmarked against 144 previously published prognostic models for LUAD, the CPM showcases enhanced prognostic accuracy.

Patients with elevated CPM scores exhibited significantly poorer prognoses in LUAD, consistently observed across seven datasets: TCGA, GSE13213, GSE26939, GSE29016, GSE30219, GSE31210 and GSE42127. The area under the curve (AUC) values for 1‐, 3‐ and 5‐year OS demonstrated the robust predictive capability of the CPM score in these datasets: TCGA (0.68, 0.68, 0.64), GSE13213 (0.95, 0.71, 0.72), GSE26939 (0.79, 0.68, 0.66), GSE29016 (0.65, 0.74, 0.70), GSE30219 (0.71, 0.81, 0.79), GSE31210 (NA, 0.75, 0.81) and GSE42127 (0.69, 0.67, 0.70) (Figure 7A–G). To further evaluate the prognostic efficacy of the CPM score, we included a panel of 144 signatures and compared the concordance index (C‐index) across the seven datasets. The results indicated that our CPM score outperformed the majority of previously published signatures in all seven datasets (Figure 7A–G).

### 3.7 Immune landscape and correlation with CPM

**FIGURE 8:**
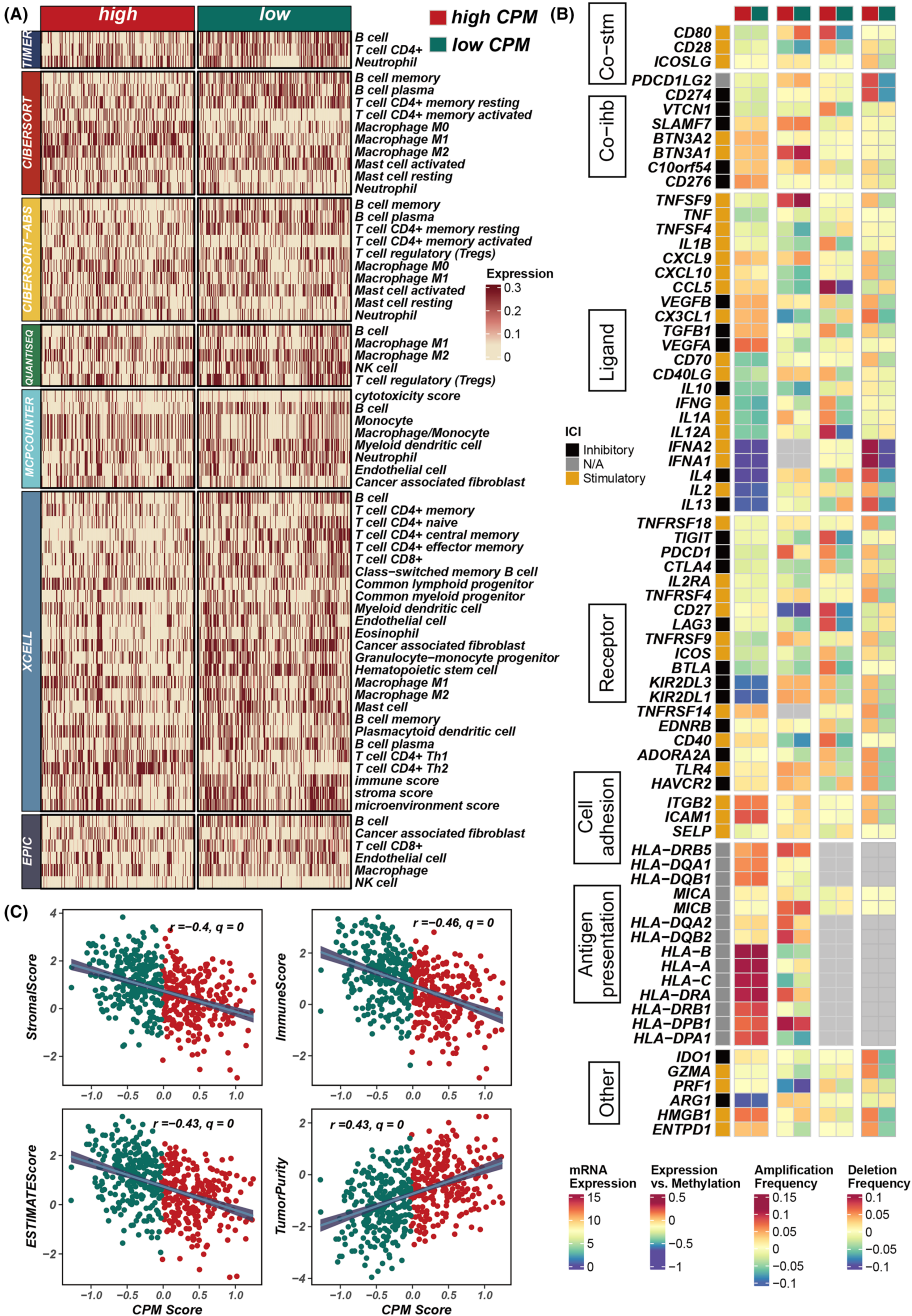
Assessment of immune infiltration and correlation with CPM scores in LUAD. (A) A heatmap illustrating the variance in immune infiltration scores between groups with high and low CPM scores. (B) Analysis depicting the relationship between CPM scores and the expression of immune‐related genes. (C) Scatter plots revealing the associations between CPM scores and various tumour microenvironment metrics, including stromal scores, immune scores, ESTIMATE scores and tumour purity.

To elucidate the immunological landscape as depicted by the CPM score, we conducted an analysis to ascertain the relationship between the CPM score and the degree of immune cell infiltration, as well as the expression of immune‐related genes. We utilized seven distinct analytical methodologies to calculate the immune infiltration scores within the TCGA dataset. Heatmap analysis revealed that a more substantial degree of immune cell infiltration was observed in cohorts with lower CPM scores (Figure 8A). Further comprehensive analysis indicated an inverse correlation between CPM scores and matrix scores, immune scores and ESTIMATE scores, which suggests stromal and immune cell presence in tumour tissue; however, a direct correlation was noted with tumour purity (Figure 8C). In assessing the association between CPM scores and immune gene expression, it was observed that the expression levels of immune‐related genes were relatively diminished in cohorts with higher CPM scores, denoting a heightened level of immune suppression in these groups (Figure 8B).

### 3.8 Validating the oncogenic role of S100A16

**FIGURE 9:**
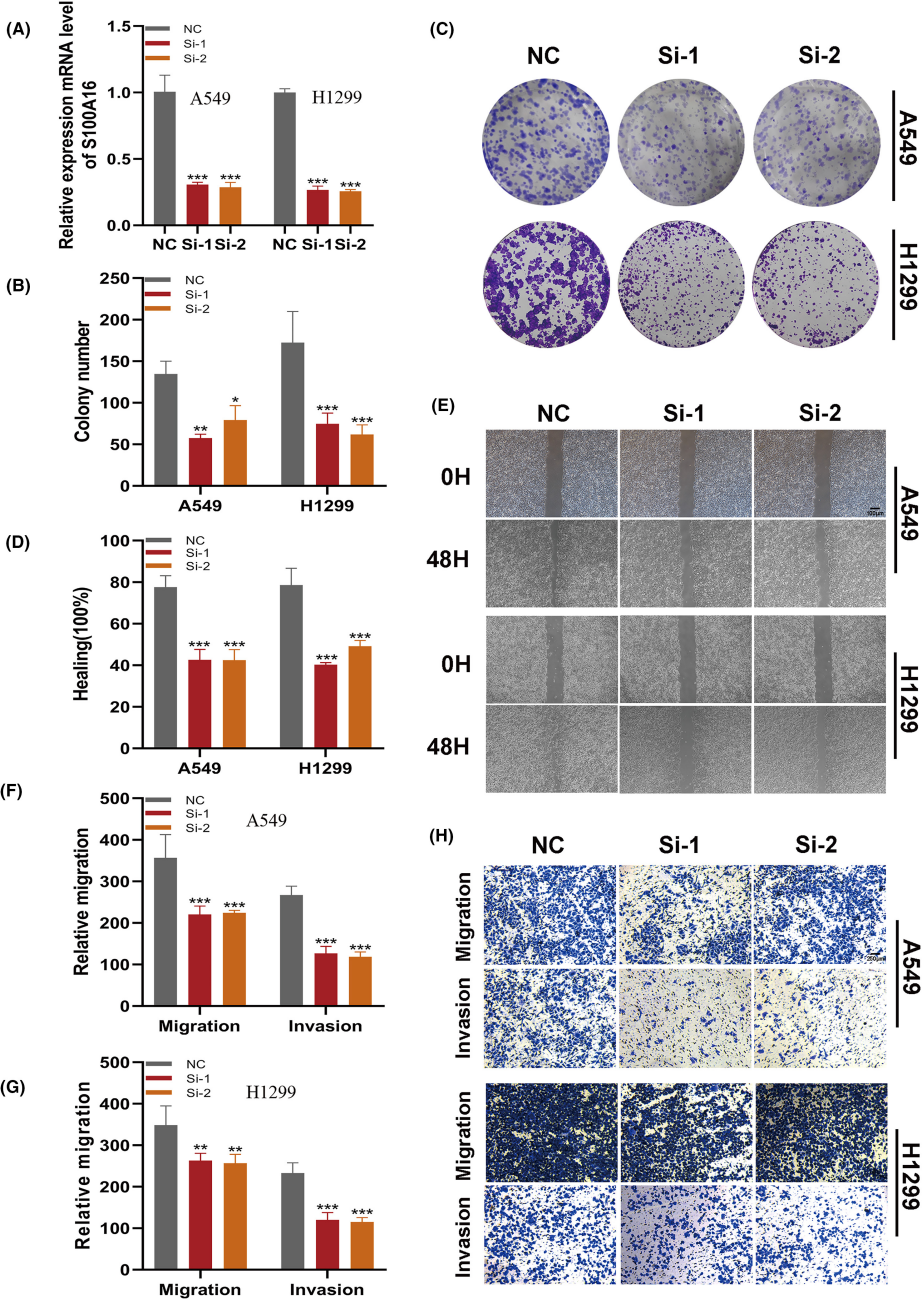
Validation of S100A16's oncogenic role in LUAD through targeted knockdown. (A) Reduction in S100A16 expression in A549 and H1299 cells post‐S100A16 knockdown. (B, C) Colony formation assays demonstrating that S100A16 knockdown notably inhibits LUAD cell proliferation. (D, E) Wound healing assays assess the migratory potential of A549 and H1299 cells following si‐S100A16 transfection. (F‐H) Transwell assays evaluate the migration and invasion capabilities of S100A16‐knockdown A549 and H1299 cells.

Among all the genes in the model, S100A16 exhibits a significant positive correlation with the model score (*R* = 0.59, *p* < 0.01, Figure S2), underscoring its prominent impact on the prognosis of LUAD. To elucidate the oncogenic role of S100A16 in LUAD, we employed siRNA to downregulate the expression of S100A16 in A549 and H1299 cell lines (Figure 9A). Colony formation assays demonstrated that suppression of S100A16 markedly inhibited the proliferation and DNA replication capabilities of LUAD cells (Figure 9B,C). Wound healing assays were conducted to assess cellular migration capacity. The findings indicated a substantial reduction in the wound closure rate of A549 and H1299 cells post‐S100A16 knockdown compared to the control group (Figure 9D,E). Furthermore, transwell assays revealed a decrease in the number of cells invading the lower chamber following S100A16 knockdown (Figure 9F–H). Collectively, these outcomes suggest a tumorigenic role for S100A16 in LUAD cells.

## 4 DISCUSSION

Based on the updated classification of LUAD, the survival rates for AAH, AIS and MIA are nearly 100%. However, as the disease progresses to IAC, the difficulty of treatment increases, along with the risk of tumour recurrence and metastasis, leading to a significant decline in treatment efficacy and survival rates.^5^, ^6^ Therefore, early diagnosis and intervention are crucial for improving the prognosis of LUAD patients, highlighting the importance of further understanding the evolutionary trajectory from precancerous lesions to invasive LUAD. Single‐cell sequencing technology allows for high‐resolution analysis of cellular origins, gene molecular specificity, immunogenicity and other aspects, dissecting the transcriptional and immunological changes associated with tumour progression, which is of significant importance in identifying biomarkers closely related to tumour progression.^16^, ^27^

In this study, we conducted extensive clustering analysis of malignant epithelial tissues in three single‐cell sequencing datasets, identifying specific cell clusters that synchronize with the evolutionary trajectory from AAH to AIS, MIA and IAC, and confirming the impact of these cell clusters on LUAD prognosis. Based on the identifying genes of the cell clusters, we constructed a prognostic prediction model for LUAD using machine learning algorithms, and validated the predictive ability of the model using multiple datasets. Subsequently, we explored the differences in immune infiltration and immune regulation between high‐ and low‐CPM groups. Finally, we experimentally validated the influence of the model gene S100A16. Based on these findings, we believe that the model constructed in this study can accurately predict the prognosis of LUAD patients.

Previous studies have shown that driver gene mutations contribute to the occurrence and development of LUAD.^28^ De Bruinet al. found that mutations in APOBEC genes are important factors influencing intratumoral heterogeneity in LUAD and contribute to the progression from AIS/MIA to IAC.^29^, ^30^ The study by Chen et al. revealed a significant increase in the mutation frequency of APOBEC, TP53 and HLA LOH from the preinvasive stage to the invasive stage, highlighting the crucial regulatory role of TP53 in LUAD invasiveness, consistent with the functional association between TP53 mutations and the invasive potential of cancer previously discovered.^31^ It is worth noting that although we have depicted the evolutionary trajectory from AAH to AIS, MIA and IAC based on high‐throughput data from LUAD samples and identified specific genes and signalling pathways involved in this process, we have not analysed the mutation frequencies of the relevant genes during the development from precancerous lesions to invasive LUAD.

S100A16, as a prognostic marker for LUAD, has been confirmed in previous studies and is closely related to the malignant transformation of tissues.^32^, ^33^, ^34^ Previous studies have found that S100A16 promotes EMT transformation of various cancer cells, including LUAD, which is an important mechanism leading to lymph node invasion and metastasis in LUAD.^35^, ^36^, ^37^, ^38^, ^39^, ^40^ Chen et al. analysed the OS data of 502 LUAD patients and found that high expression of S100A16 is associated with poorer OS.^41^ Li et al. constructed a prognostic model for LUAD based on seven immune hypoxia‐related genes, including S100A16, S100P, PGK1, TNFSF11, ARRB1, NCR3 and TSLP, which can evaluate the immune status and predict the prognosis of LUAD patients.^42^ In this study, we found high expression of S100A16 in LUAD, which indicates a shorter OS. Knocking down the S100A16 gene significantly inhibited the proliferation, invasion, and DNA replication ability of LUAD cells. Therefore, we believe that S100A16 plays a critical role in the progression of LUAD.

However, this study also has some limitations. Firstly, the biological mechanisms by which model genes influence the progression of LUAD from precancerous lesions to invasive cancer need further in‐depth research. Second, the specific presentation of mutation frequencies of relevant genes during tumour progression was not analysed. Third, the model requires more in vitro experiments and clinical validation.

In summary, our investigation provides a comprehensive molecular and functional characterization of tumour cell heterogeneity in LUAD. Through integrating insights from cell classification, CNV analysis, dynamic gene expression profiling, pathway enrichment studies, and machine learning‐derived CPM, we offer a nuanced understanding of cancer biology complexities and their implications for prognosis and therapeutic interventions. This integrated approach not only advances our knowledge of LUAD but also lays the groundwork for the development of more effective diagnostic and therapeutic approaches, ultimately enhancing personalized oncology and improving patient care outcomes.

## AUTHOR CONTRIBUTIONS

**Pengpeng Zhang:** Conceptualization (equal); supervision (equal); writing – review and editing (equal). **Jiaqi Feng:** Conceptualization (equal); data curation (equal); supervision (equal). **Min Rui:** Data curation (equal); formal analysis (equal); visualization (equal). **Jiping Xie:** Investigation (equal); writing – review and editing (equal). **Lianmin Zhang:** Funding acquisition (equal); writing – original draft (equal). **Zhenfa Zhang:** Funding acquisition (equal); project administration (equal); writing – review and editing (equal).

## FUNDING INFORMATION

This work was supported by the Tianjin Natural Science Foundation under Grant/Award Number 21JCYBJC01020.

## CONFLICT OF INTEREST STATEMENT

It is hereby declared by the authors that the research was carried out without the presence of any potential conflict of interest arising from commercial or financial relationships.

## Supporting information


Figure S1.

Figure S2.

## Data Availability

The datasets analysed in the current study are available in the TCGA repository (http://cancergenome.nih.gov/), and GEO (https://www. ncbi.nlm.nih.gov/geo/).

## References

[1] Sung H , Ferlay J , Siegel RL , et al. Global cancer statistics 2020: GLOBOCAN estimates of incidence and mortality worldwide for 36 cancers in 185 countries. CA Cancer J Clin. 2021;3:209‐249. 10.3322/caac.21660 33538338

[2] Thai AA , Solomon BJ , Sequist LV , Gainor JF , Heist RS . Lung cancer. Lancet. 2021;398(10299):535‐554. 34273294 10.1016/S0140-6736(21)00312-3

[3] Schabath MB , Cote ML . Cancer progress and priorities: lung cancer. Cancer Epidemiol Biomarkers Prev. 2019;28(10):1563‐1579. 31575553 10.1158/1055-9965.EPI-19-0221 PMC6777859

[4] Yoshizawa A , Motoi N , Riely GJ , et al. Impact of proposed IASLC/ATS/ERS classification of lung adenocarcinoma: prognostic subgroups and implications for further revision of staging based on analysis of 514 stage I cases. Mod Pathol. 2011;24(5):653‐664. 21252858 10.1038/modpathol.2010.232

[5] Travis WD , Brambilla E , Noguchi M , et al. International association for the study of lung cancer/american thoracic society/european respiratory society international multidisciplinary classification of lung adenocarcinoma. J Thorac Oncol. 2011;6(2):244‐285. 21252716 10.1097/JTO.0b013e318206a221 PMC4513953

[6] Woo T , Okudela K , Mitsui H , et al. Prognostic value of the IASLC/ATS/ERS classification of lung adenocarcinoma in stage I disease of Japanese cases. Pathol Int. 2012;62(12):785‐791. 23252867 10.1111/pin.12016

[7] Church TR , Black WC , Aberle DR , et al. Results of initial low‐dose computed tomographic screening for lung cancer. N Engl J Med. 2013;368(21):1980‐1991. 23697514 10.1056/NEJMoa1209120 PMC3762603

[8] Walts AE , Marchevsky AM . Root cause analysis of problems in the frozen section diagnosis of in situ, minimally invasive, and invasive adenocarcinoma of the lung. Arch Pathol Lab Med. 2012;136(12):1515‐1521. 23194044 10.5858/arpa.2012-0042-OA

[9] Yeh Y‐C , Nitadori J‐i , Kadota K , et al. Using frozen section to identify histological patterns in stage I lung adenocarcinoma of ≤3 cm: accuracy and interobserver agreement. Histopathology. 2015;66(7):922‐938. 24889415 10.1111/his.12468 PMC4536823

[10] Dutta AK , Alberge JB , Sklavenitis‐Pistofidis R , Lightbody ED , Getz G , Ghobrial IM . Single‐cell profiling of tumour evolution in multiple myeloma–opportunities for precision medicine. Nat Rev Clin Oncol. 2022;19(4):223‐236. 35017721 10.1038/s41571-021-00593-y

[11] Gohil SH , Iorgulescu JB , Braun DA , Keskin DB , Livak KJ . Applying high‐dimensional single‐cell technologies to the analysis of cancer immunotherapy. Nat Rev Clin Oncol. 2021;18(4):244‐256. 33277626 10.1038/s41571-020-00449-x PMC8415132

[12] de Sousa VML , Carvalho L . Heterogeneity in lung cancer. Pathobiology. 2018;85(1‐2):96‐107. 29635240 10.1159/000487440

[13] Wang C , Yu Q , Song T , et al. The heterogeneous immune landscape between lung adenocarcinoma and squamous carcinoma revealed by single‐cell RNA sequencing. Signal Transduct Target Ther. 2022;7(1):289. 36008393 10.1038/s41392-022-01130-8 PMC9411197

[14] Hu X , Fujimoto J , Ying L , et al. Multi‐region exome sequencing reveals genomic evolution from preneoplasia to lung adenocarcinoma. Nat Commun. 2019;10(1):2978. 31278276 10.1038/s41467-019-10877-8 PMC6611767

[15] Li Y , Li X , Li H , et al. Genomic characterisation of pulmonary subsolid nodules: mutational landscape and radiological features. Eur Respir J. 2020;55(2):1901409. 31699841 10.1183/13993003.01409-2019

[16] Hwang B , Lee JH , Bang D . Single‐cell RNA sequencing technologies and bioinformatics pipelines. Exp Mol Med. 2018;50(8):1‐14. 10.1038/s12276-018-0071-8 PMC6082860 30089861

[17] Tomida S , Takeuchi T , Shimada Y , et al. Relapse‐related molecular signature in lung adenocarcinomas identifies patients with dismal prognosis. J Clin Oncol. 2009;27(17):2793‐2799. 19414676 10.1200/JCO.2008.19.7053

[18] Wilkerson MD , Yin X , Walter V , et al. Differential pathogenesis of lung adenocarcinoma subtypes involving sequence mutations, copy number, chromosomal instability, and methylation. PLoS One. 2012;7(5):e36530. 22590557 10.1371/journal.pone.0036530 PMC3349715

[19] Staaf J , Jönsson G , Jönsson M , et al. Relation between smoking history and gene expression profiles in lung adenocarcinomas. BMC Med Genet. 2012;5:22. 10.1186/1755-8794-5-22 PMC3447685 22676229

[20] Rousseaux S , Debernardi A , Jacquiau B , et al. Ectopic activation of germline and placental genes identifies aggressive metastasis‐prone lung cancers. Sci Transl Med. 2013;5(186):186ra66. 10.1126/scitranslmed.3005723 PMC4818008 23698379

[21] Okayama H , Kohno T , Ishii Y , et al. Identification of genes upregulated in ALK‐positive and EGFR/KRAS/ALK‐negative lung adenocarcinomas. Cancer Res. 2012;72(1):100‐111. 22080568 10.1158/0008-5472.CAN-11-1403

[22] Tang H , Xiao G , Behrens C , et al. A 12‐gene set predicts survival benefits from adjuvant chemotherapy in non‐small cell lung cancer patients. Clin Cancer Res. 2013;19(6):1577‐1586. 23357979 10.1158/1078-0432.CCR-12-2321 PMC3619002

[23] Gulati GS , Sikandar SS , Wesche DJ , et al. Single‐cell transcriptional diversity is a hallmark of developmental potential. Science. 2020;367(6476):405‐411. 31974247 10.1126/science.aax0249 PMC7694873

[24] Wu Y , Yang S , Ma J , et al. Spatiotemporal immune landscape of colorectal cancer liver metastasis at single‐cell level. Cancer Discov. 2022;12(1):134‐153. 34417225 10.1158/2159-8290.CD-21-0316

[25] Hänzelmann S , Castelo R , Guinney J . GSVA: gene set variation analysis for microarray and RNA‐seq data. BMC Bioinformatics. 2013;14:7. 23323831 10.1186/1471-2105-14-7 PMC3618321

[26] Yoshihara K , Shahmoradgoli M , Martínez E , et al. Inferring tumour purity and stromal and immune cell admixture from expression data. Nat Commun. 2013;4:2612. 24113773 10.1038/ncomms3612 PMC3826632

[27] Sklavenitis‐Pistofidis R , Getz G , Ghobrial I . Single‐cell RNA sequencing: one step closer to the clinic. Nat Med. 2021;27(3):375‐376. 33664491 10.1038/s41591-021-01276-y

[28] Murphy SJ , Wigle DA , Lima JF , et al. Genomic rearrangements define lineage relationships between adjacent lepidic and invasive components in lung adenocarcinoma. Cancer Res. 2014;74(11):3157‐3167. 24879567 10.1158/0008-5472.CAN-13-1727 PMC4399556

[29] de Bruin EC , McGranahan N , Mitter R , et al. Spatial and temporal diversity in genomic instability processes defines lung cancer evolution. Science (New York, NY). 2014;346(6206):251‐256. 10.1126/science.1253462 PMC4636050 25301630

[30] Vinayanuwattikun C , Le Calvez‐Kelm F , Abedi‐Ardekani B , et al. Elucidating genomic characteristics of lung cancer progression from in situ to invasive adenocarcinoma. Sci Rep. 2016;6:31628. 27545006 10.1038/srep31628 PMC4992872

[31] Chen H , Carrot‐Zhang J , Zhao Y , et al. Genomic and immune profiling of pre‐invasive lung adenocarcinoma. Nat Commun. 2019;10(1):5472. 31784532 10.1038/s41467-019-13460-3 PMC6884501

[32] Wu C , Yang J , Lin X , Wu J , Yang C , Chen S . LncRNA PRKCA‐AS1 promotes LUAD progression and function as a ceRNA to regulate S100A16 by sponging miR‐508‐5p. J Cancer. 2024;15(6):1718‐1730. 38370382 10.7150/jca.91184 PMC10869986

[33] Zhu J , Wang M , Hu D . Identification of prognostic immune‐related genes by integrating mRNA expression and methylation in lung adenocarcinoma. Int j Genomics. 2020;2020:9548632. 32695805 10.1155/2020/9548632 PMC7368195

[34] Basnet S , Vallenari EM , Maharjan U , Sharma S , Schreurs O , Sapkota D . An update on S100A16 in human cancer. Biomolecules. 2023;13(7):1070. 37509106 10.3390/biom13071070 PMC10377057

[35] Zhou Y , Zhang Z , Wang N , et al. Suppressor of cytokine signalling‐2 limits IGF1R‐mediated regulation of epithelial‐mesenchymal transition in lung adenocarcinoma. Cell Death Dis. 2018;9(4):429. 29559623 10.1038/s41419-018-0457-5 PMC5861121

[36] You X , Li M , Cai H , et al. Calcium binding protein S100A16 expedites proliferation, invasion and epithelial‐mesenchymal transition process in gastric cancer. front cell. Dev Biol. 2021;9:736929. 10.3389/fcell.2021.736929 PMC8505768 34650982

[37] Wu C , Yang J , Lin X , Li R , Wu J . miR‐508‐5p serves as an anti‐oncogene by targeting S100A16 to regulate AKT signaling and epithelial‐mesenchymal transition process in lung adenocarcinoma cells. Am J Med Sci. 2023;365(6):520‐531. 36967030 10.1016/j.amjms.2023.02.014

[38] Ou S , Liao Y , Shi J , et al. S100A16 suppresses the proliferation, migration and invasion of colorectal cancer cells in part via the JNK/p38 MAPK pathway. Mol Med Rep. 2021;23(2):164. 33355370 10.3892/mmr.2020.11803 PMC7789101

[39] Lv H , Hou H , Lei H , et al. MicroRNA‐6884‐5p regulates the proliferation, invasion, and EMT of gastric cancer cells by directly targeting S100A16. Oncol Res. 2020;28(3):225‐236. 31796150 10.3727/096504019X15753718797664 PMC7851531

[40] Li T , Ren T , Huang C , et al. S100A16 induces epithelial‐mesenchymal transition in human PDAC cells and is a new therapeutic target for pancreatic cancer treatment that synergizes with gemcitabine. Biochem Pharmacol. 2021;189:114396. 33359364 10.1016/j.bcp.2020.114396

[41] Chen LL , Liang C . Aberrant S100A16 expression might be an independent prognostic indicator of unfavorable survival in non‐small cell lung adenocarcinoma. PLoS One. 2018;13(5):e0197402. 29746588 10.1371/journal.pone.0197402 PMC5945035

[42] Li Y , Huang H , Jiang M , et al. Identification and validation of a hypoxia‐immune signature for overall survival prediction in lung adenocarcinoma. Front Genet. 2022;13:975279. 36263421 10.3389/fgene.2022.975279 PMC9573950

