# Supplementary figures and images for: Integrating machine learning and single‐cell analysis to uncover lung adenocarcinoma progression and prognostic biomarkers
Source: J Cell Mol Med. 2024 Jul 3;28(13):e18516. doi: 10.1111/jcmm.18516 (PMC11221317; doi:10.1111/jcmm.18516)

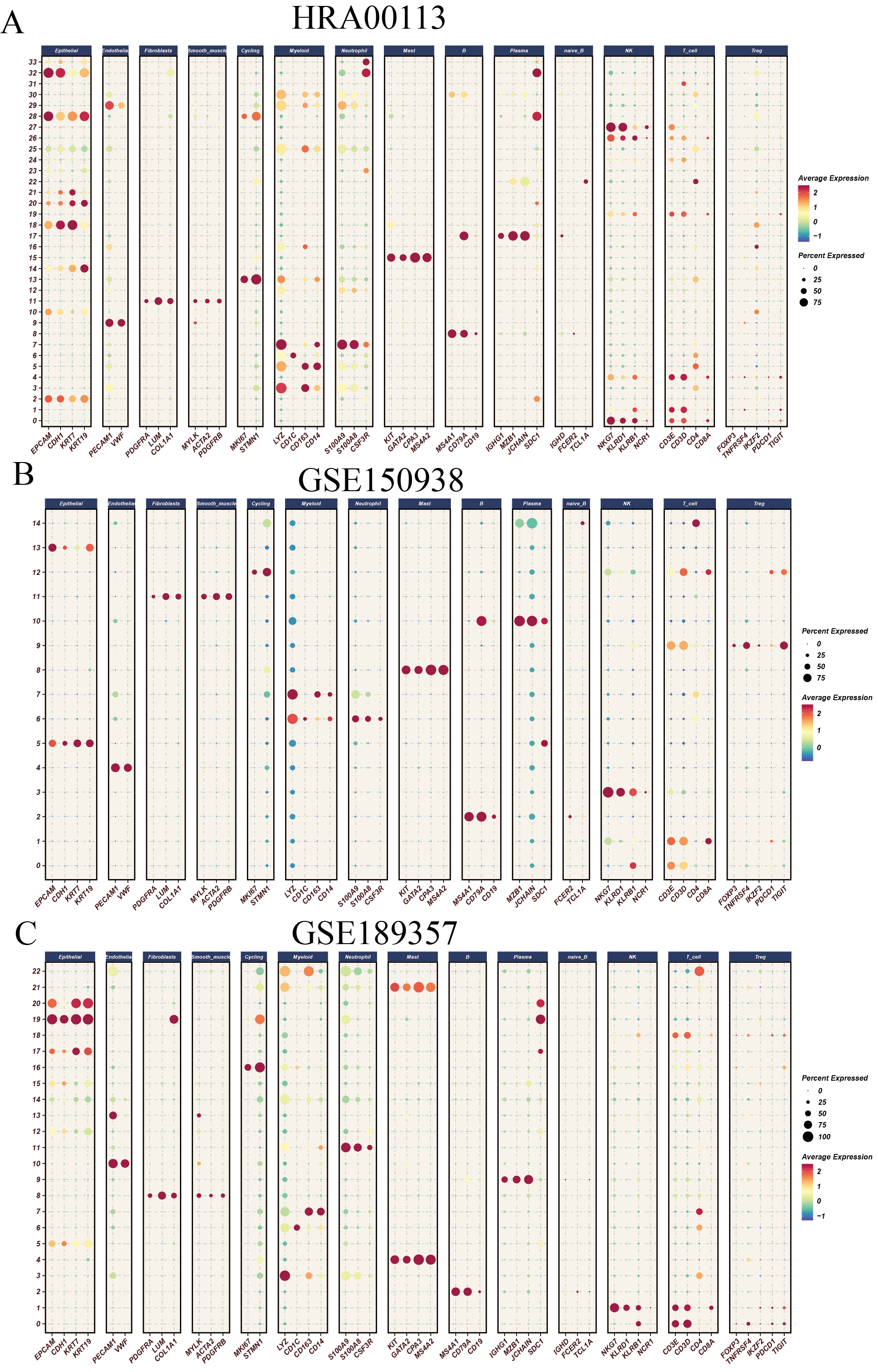

Supplement: Supplementary file 1 — Figure S1. [file JCMM-28-e18516-s001.tif]

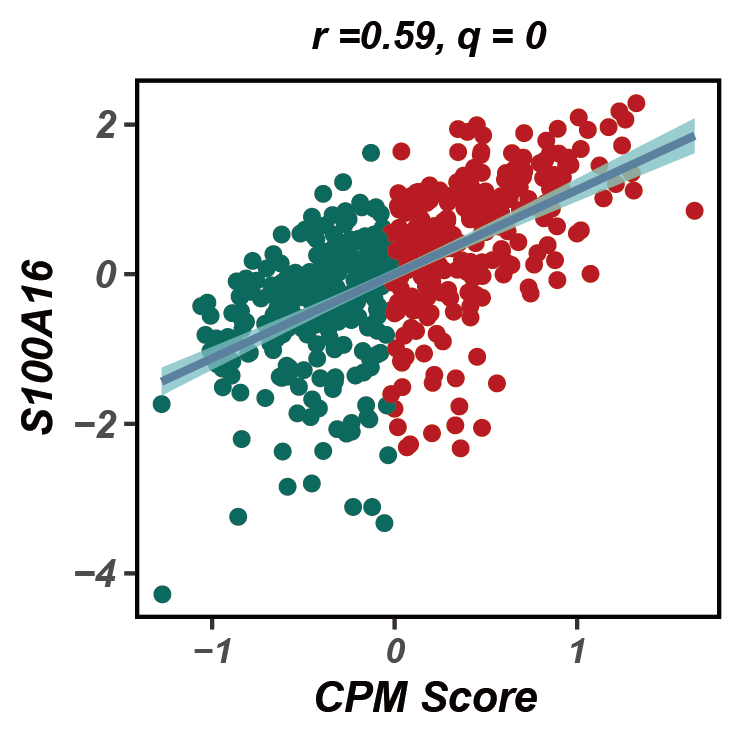

Supplement: Supplementary file 2 — Figure S2. [file JCMM-28-e18516-s002.tif]
